# Antimicrobial Activity of Ceftolozane-Tazobactam, Ceftazidime-Avibactam, and Cefiderocol against Multidrug-Resistant Pseudomonas aeruginosa Recovered at a German University Hospital

**DOI:** 10.1128/spectrum.01697-22

**Published:** 2022-10-03

**Authors:** C. Weber, T. Schultze, S. Göttig, J. Kessel, A. Schröder, M. Tietgen, S. Besier, T. Burbach, S. Häussler, T. A. Wichelhaus, D. Hack, V. A. J. Kempf, M. Hogardt

**Affiliations:** a Institute for Medical Microbiology and Infection Control, University Hospital Frankfurt, Goethe University, Frankfurt am Main, Germany; b University Center of Competence for Infection Control of the State of Hesse, Frankfurt am Main, Germany; c Department of Internal Medicine, Infectious Diseases, University Hospital Frankfurt, Goethe University, Frankfurt am Main, Germany; d German National Consiliary Laboratory on Cystic Fibrosis Bacteriology, Frankfurt am Main, Germany; e Department of Molecular Bacteriology, Helmholtz Center for Infection Research, Braunschweig, Germany; f Department of Clinical Microbiology, Copenhagen University Hospital - Rigshospitalet, Copenhagen, Denmark; University of Manitoba

**Keywords:** multidrug resistance, *Pseudomonas aeruginosa*, high-risk clones, ceftolozane-tazobactam, ceftazidime-avibactam, cefiderocol, carbapenemases

## Abstract

Multidrug-resistant (MDR) Pseudomonas aeruginosa increasingly causes health care-associated infections. In this study, we determined the activity of ceftolozane-tazobactam, ceftazidime-avibactam, and cefiderocol against 223 MDR P. aeruginosa clinical isolates recovered from 2013 to 2017 at the University Hospital Frankfurt by using MIC test strips. Furthermore, we evaluated the presence of genes encoding major β-lactamases, such as VIM, IMP, NDM, GIM, SPM, and KPC; the extended spectrum β-lactamase (ESBL)-carbapenemase GES; and the virulence-associated traits ExoS and ExoU, as in particular ExoU is thought to be associated with poor clinical outcome. For MDR P. aeruginosa isolates, the MIC_50_/MIC_90_ values of ceftolozane-tazobactam, ceftazidime-avibactam, and cefiderocol were 8/>256 mg/L, 16/>256 mg/L, and 0.25/1 mg/L, respectively. Cefiderocol showed the highest susceptibility rate (97.3%) followed by ceftazidime-avibactam (48.4%) and ceftolozane-tazobactam (46.6%). In 81 (36.3%) isolates, carbapenemase gene *bla*_VIM_ was detected, and in 5 (2.2%) isolates, *bla*_GES_ was detected (with a positive association of *exoU* and *bla*_VIM_). More than half of the isolates belong to the so-called international P. aeruginosa “high-risk” clones, with sequence type 235 (ST235) (24.7%) being the most prevalent. This study underlines that ceftolozane-tazobactam, ceftazidime-avibactam, and cefiderocol are important options for the treatment of infections due to MDR P. aeruginosa, with cefiderocol currently being the most active available antipseudomonal β-lactam agent. According to our clinical experience, the outcome of cefiderocol therapy (8 patients) was favorable especially in cases of MDR P. aeruginosa-associated complicated urinary tract infections.

**IMPORTANCE** After testing ceftolozane-tazobactam, ceftazidime-avibactam, and cefiderocol against a collection of 233 multidrug-resistant (MDR) Pseudomonas aeruginosa, we showed that cefiderocol is the most active antipseudomonal β-lactam agent (susceptibility rates were 46.6%, 48.4%, and 97.4%, respectively). The most prevalent one was sequence type 235 (ST235) (24.7%), followed by ST244, ST175, and ST233, with all belonging to the top 10 P. aeruginosa high-risk clones with worldwide distribution. Our data indicate that during surveillance studies special attention should be paid to the MDR and highly virulent VIM- and ExoU-producing variant of ST235. Furthermore, in the case of infections caused by carbapenemase-producing MDR P. aeruginosa, cefiderocol is the preferred treatment option, while outcomes of complicated urinary tract infections and hospital-acquired pneumonia with cefiderocol were favorable.

## INTRODUCTION

Pseudomonas aeruginosa is a ubiquitously distributed opportunistic Gram-negative bacterium that commonly causes severe health care-associated infections in compromised patients. P. aeruginosa belongs to the ESKAPE organisms including Enterococcus faecium, Staphylococcus aureus, Klebsiella pneumoniae, Acinetobacter baumannii, P. aeruginosa, and Enterobacter that are of major clinical importance and is thus one of the critical pathogens with an urgent need for the development of new antibiotics (1).

Apart from its high intrinsic resistance, P. aeruginosa possesses an outstanding capacity to develop resistance to nearly all available antibiotics (2). Infections due to multidrug-resistant (MDR) or even extensively drug-resistant (XDR) P. aeruginosa are associated with a significant increase in morbidity and mortality (3). The spread of P. aeruginosa high-risk clones is thought to play a major role in the global increase in MDR/XDR phenotypes (4, 5). The availability of novel β-lactam, β-lactamase inhibitor combinations, such as ceftolozane-tazobactam, ceftazidime-avibactam, and the recent introduction of cefiderocol, has partially alleviated the urgent clinical need for new agents to combat infections by MDR/XDR P. aeruginosa (6).

Ceftolozane-tazobactam is the combination of the new third-generation cephalosporin ceftolozane and the known β-lactamase inhibitor tazobactam. Ceftolozane has improved activity against P. aeruginosa relative to most other β-lactams, as it is stable against AmpC enzymes and also a poor inducer of *ampC* expression (7). Ceftolozane-tazobactam is not affected by active efflux and not affected by OprD porin alterations but exhibits no reliable activity against producers of carbapenemases, such as class B metallo-β-lactamases (MBLs) and class A and class D serine β-lactamases (8).

Ceftazidime-avibactam is a combination of ceftazidime with the new non-β-lactam β-lactamase inhibitor avibactam that improves the antimicrobial activity of ceftazidime against MDR Gram-negative organisms carrying extended spectrum β-lactamases (ESBLs) (e.g., TEM-, SHV-, and CTX-M-type), KPC carbapenemases, AmpC cephalosporinases, and some class D β-lactamases (e.g., OXA-48). Ceftazidime-avibactam is not active against isolates producing MBLs.

Ceftolozane-tazobactam and ceftazidime-avibactam have been approved for complicated urinary tract infections (cUTIs), complicated intra-abdominal infections (cIAIs), and hospital-acquired pneumonia (HAP), including ventilator-associated pneumonia (VAP) (8).

Cefiderocol is a novel catechol-substituted siderophore cephalosporin that uses bacterial iron transporters (“Trojan horse” strategy) to pass the outer membrane into the periplasm. This novel mechanism of periplasmic entry via active iron transport systems overcomes classical β-lactam resistance mechanisms. Thus, cefiderocol possesses a potent activity against MDR Gram-negative bacteria, including carbapenem-resistant organisms (9). This broad activity results partly from its high stability against various ESBLs, serine-type carbapenemases, and MBLs. In P. aeruginosa, the iron receptor-dependent uptake of cefiderocol may overcome β-lactam resistance associated with OprD porin deficiency and AmpC overexpression. Cefiderocol is approved for the treatment of cUTIs, as well as HAP/VAP, due to Gram-negative bacteria in case of limited treatment options (10, 11).

Regarding acquired β-lactam resistance among MDR P. aeruginosa, the emergence and spread of carbapenemases is of major epidemiological concern. MBLs are the most prevalent type of carbapenemases produced by P. aeruginosa, while VIMs (mainly VIM-1 and VIM-2) are the most prevalent ones followed by IMPs. Other MBLs (such as NDM, GIM, and SPM) and KPC- (class A serine β-lactamase) and OXA-type (class D serine β-lactamases) β-lactamases are identified rarely among P. aeruginosa (12). Furthermore, the class A ESBL GES is common in P. aeruginosa. GES enzymes are notable due to their ability to expand their spectrum to carbapenems because of amino acid substitutions (13).

This study compared the activity of ceftolozane-tazobactam, ceftazidime-avibactam, and cefiderocol against clinical MDR/XDR isolates of P. aeruginosa. To also elucidate the epidemiological situation of MDR/XDR P. aeruginosa in our hospital and to put these data into an international context, we performed multilocus sequence typing (MLST). Furthermore, we determined the presence of the most relevant carbapenemase genes (*bla*_VIM_, *bla*_IMP_, *bla*_GES_, *bla*_NDM_, *bla*_GIM_, *bla*_SPM_, and *bla*_KPC_) and that of the type III secretion system-dependent virulence genes *exoS* (encoding ADP-ribosyltransferase exotoxin S) and *exoU* (encoding phospholipase A_2_ exotoxin U) that are important virulence and epidemiological traits of P. aeruginosa population structure, as ExoS and ExoU are mutually exclusively found in P. aeruginosa (14, 15). In particular ExoU is an interesting target for surveillance studies, as it is highly cytotoxic, associated with poor clinical outcome, and associated with the spread of sequence type 235 (ST235), which is the most common one among the top 10 globally distributed P. aeruginosa high-risk clones (4). Finally, we report on our experience regarding the clinical efficacy of cefiderocol in MDR P. aeruginosa infections.

## RESULTS

A total of 223 MDR P. aeruginosa clinical isolates from 2013 to 2017 were evaluated for susceptibility to ceftolozane-tazobactam, ceftazidime-avibactam, and cefiderocol by using MIC test strips (MTS). Isolates were derived from respiratory secretions (*n* = 50), wound swabs (*n* = 26), urine (*n* = 24), medical devices (*n* = 7), stool samples (*n* = 7), tissue biopsy specimens (*n* = 6), blood cultures (*n* = 4), urogenital samples (*n* = 3), and other origins (*n* = 7). Additionally, 89 hygiene screening samples (rectal, skin, and oropharyngeal swabs) were included. Seventy-six isolates were collected from medical wards, 60 isolates from intensive care units (ICUs), 40 isolates from surgical wards, 28 isolates from hematology wards, 3 isolates from pediatric wards, and 16 isolates from other origins. Among 223 MDR P. aeruginosa, 84.3% were additionally nonsusceptible to aztreonam, 54.3% to tobramycin, 42.6% to fosfomycin, and 38.1% to amikacin (data not shown). All isolates were susceptible to colistin. Thus, 71% of isolates represent XDR P. aeruginosa according to the definition of Magiorakos et al. (16).

### Antimicrobial activity of ceftolozane-tazobactam, ceftazidime-avibactam, and cefiderocol against MDR P. aeruginosa.

**FIG 1:**
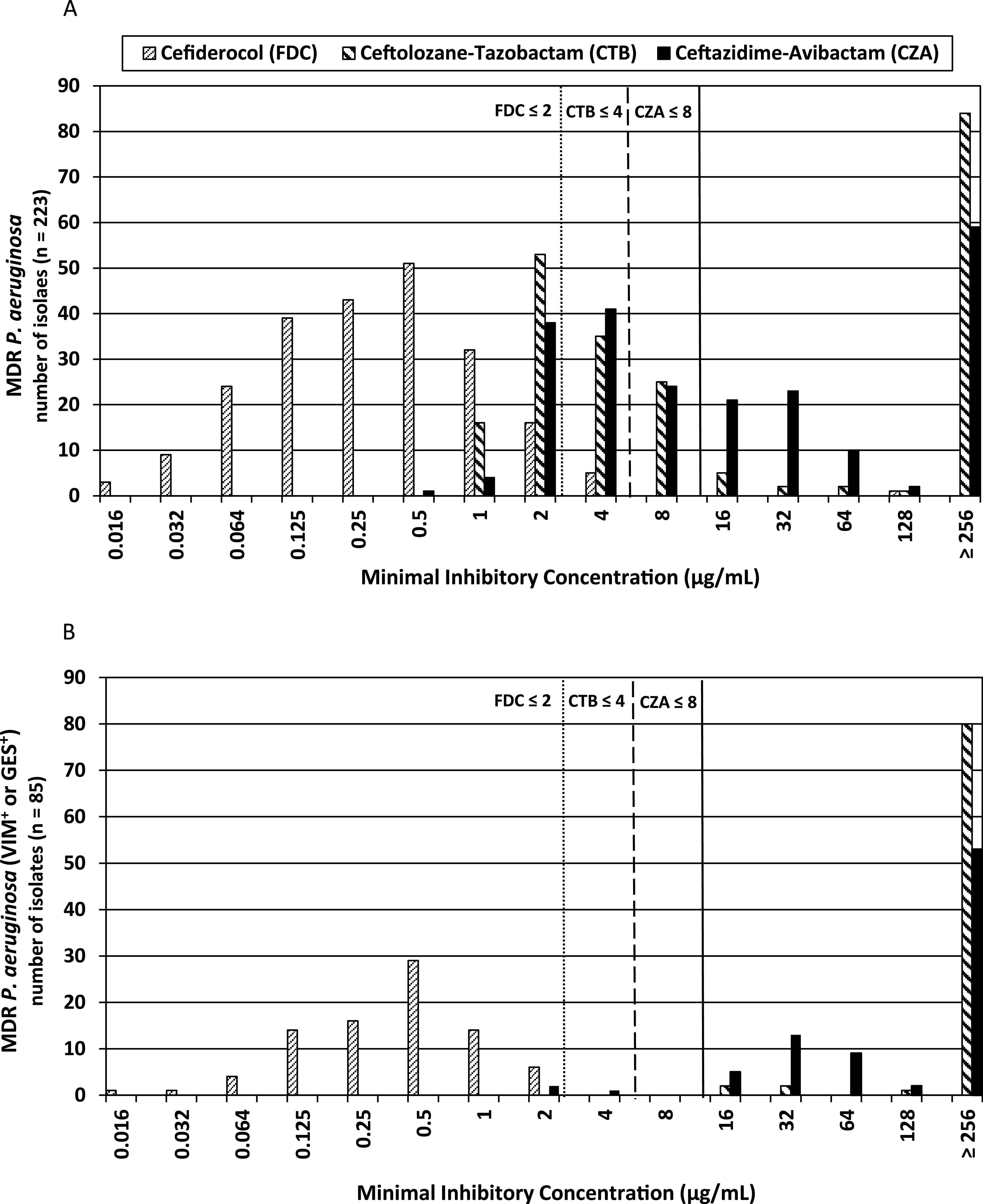
(A and B) MIC distributions of ceftolozane-tazobactam, ceftazidime-avibactam, and cefiderocol among tested MDR P. aeruginosa (*n* = 223) (A) and all carbapenemase-producing MDR P. aeruginosa (*n* = 85) (B). EUCAST susceptibility breakpoints are indicated.

Of 223 tested MDR P. aeruginosa isolates, 53.4% (119/223) were resistant to ceftolozane-tazobactam, 51.6% (115/223) were resistant to ceftazidime-avibactam, and 2.7% (6/223) were resistant to cefiderocol as determined by MTS. Resistance rates, MIC_50_, MIC_90_, and MIC range of MDR P. aeruginosa are given in Table 1. In contrast to cefiderocol, MIC distributions for ceftazidime-avibactam and ceftolozane-tazobactam did not follow a Gaussian distribution as found for wild-type populations (Fig. 1A). Cefiderocol was the most active substance, followed by ceftazidime-avibactam and ceftolozane-tazobactam. Of note, less than 50% of MDR P. aeruginosa remained susceptible to ceftazidime-avibactam or ceftolozane-tazobactam, while cefiderocol was active against 97.3% of isolates. Eighty-seven (39%) isolates were susceptible, and 100 (44.8%) isolates were resistant to both ceftolozane-tazobactam and ceftazidime-avibactam.

**TABLE 1:** Antimicrobial activity of ceftolozane-tazobactam, ceftazidime-avibactam, and cefiderocol against 223 MDR P. aeruginosa isolates^*a*^

Antimicrobial agent by organism	MIC_50_ (mg/L)	MIC_90_ (mg/L)	MIC range (mg/L)	%S	%R
P. aeruginosa MDR (*n* = 223)					
Ceftolozane-tazobactam	8	>256	1 to >256	46.6	53.4
Ceftazidime-avibactam	16	>256	0.5 to >256	48.4	51.6
Cefiderocol	0.25	1	0.016 to 128	97.3	2.7
P. aeruginosa MDR (*n* = 85) (carbapenemase positive^*b*^)					
Ceftolozane-tazobactam	>256	>256	16 to >256	0	100
Ceftazidime-avibactam	>256	>256	2 to >256	3.5^*c*^	96.5
Cefiderocol	0.5	1	0.016 to 2	100	0
P. aeruginosa MDR (*n* = 138) (carbapenemase negative^*d*^)					
Ceftolozane-tazobactam	2	8	1 to >256	75.4	24.6
Ceftazidime-avibactam	4	32	0.5 to >256	76.1	23.9
Cefiderocol	0.25	2	0.016 to 128	95.7	4.3

a From University Hospital Frankfurt in Germany from 2013 to 2017.

b Including 81 VIM-producing and 4 GES-5 producing isolates.

c Represented by 3 GES-5-producing isolates.

d Including one isolate positive for ESBL-GES-7.

For quality-control (QC) reasons, we compared the performance of the recently approved cefiderocol MTS with agar dilution. This methodological comparison showed a categorical agreement of 96% and an essential agreement of 85% (*n* = 193) (data not shown). Finally, 31 isolates failed to meet the criterion for essential agreement. MICs determined by agar dilution tend to be lower than those determined by MTS (19 out of 31). Error rates for cefiderocol MTS were as follows: for very major errors, 67% (10/15); and for major errors, 0.5% (*n* = 1/208). MIC values of isolates (*n* = 10) that account for very major errors for cefiderocol were all close to the EUCAST breakpoint (average MICs for MTS and agar dilution were 1.8 and 5.2). Due to the high very major error rate, we retested cefiderocol-resistant isolates by broth microdilution (BMD). Finally, using BMD, we confirmed only 2 isolates as truly cefiderocol resistant, resulting in no very major errors for MTS and a corrected major error rate of 1.8% (*n* = 4/221). These data indicate that MTS are a reliable method to determine cefiderocol susceptibility of MDR/XDR P. aeruginosa.

### Prevalence of carbapenemases among MDR P. aeruginosa.

PCR analyses were performed for the detection of genes encoding VIM and IMP MBLs and class A ESBL/carbapenemase GES. In total, 81 out of 223 isolates were found to be positive for *bla*_VIM_ and 5 isolates for the *bla*_GES_ gene, while no *bla*_IMP_-positive isolates were identified. Furthermore, using whole-genome sequencing (WGS) data beside IMP, we did not detect GIM, NDM, SPM, and KPC that are already rare in P. aeruginosa. Sequencing of GES β-lactamase genes showed that GES-producing isolates harbored GES-5 (*n* = 4), which is known to exhibit carbapenemase activity, and GES-7 (*n* = 1), which is known for its ESBL activity. VIM MBLs belonged to subtypes VIM-1 (*n* = 36) and VIM-2 (*n* = 45). Resistance rates, MIC_50_, MIC_90_, and the MIC range of carbapenemase-producing isolates are given in Table 1. Coresistance for ceftolozane-tazobactam and ceftazidime-avibactam was found in 96.5% of carbapenemase-producing isolates. All VIM-producing isolates were resistant to both ceftolozane-tazobactam and ceftazidime-avibactam, while 3 out of 5 GES-producing isolates were susceptible to ceftazidime-avibactam but resistant to ceftolozane-tazobactam (all 3 harbored GES-5). In contrast, all carbapenemase-producing isolates were susceptible to cefiderocol (Fig. 1B).

### Presence of pathogenicity marker genes *exoS* and *exoU* among MDR P. aeruginosa.

In addition to multidrug resistance, the individual virulence potential of P. aeruginosa may be one reason for a poor clinical outcome. Therefore, we also determined the presence of the genes encoding type III secretion system exotoxins ExoS and ExoU, as especially ExoU is considered responsible for a highly cytotoxic phenotype (17). In total, the *exoS* or *exoU* gene was detected in 147/223 (65.9%) and 74/223 (33.2%) of MDR P. aeruginosa isolates, respectively. Finally, 221 of 223 isolates (99.1%) carried either the *exoS* or *exoU* gene. Two P. aeruginosa isolates were found to be negative for both *exoS* and *exoU* genes. Interestingly, the *exoU ^+^* isolates showed with 59.5% (44/74) a higher proportion of *bla*_(VIM)_ positivity than the *exoS ^+^* isolates with only 25.2% (37/147) (chi-square test; *P* < 0.05). In contrast, all *bla*_(GES)_*^+^* isolates were of *exoS* ^−^/*exoU ^+^* genotype.

### Multilocus sequence typing (MLST) and extended resistance gene analysis.

Whole-genome sequencing revealed 44 different sequence types with 24.7% of isolates to ST235, 11.2% to ST244, 9.9% in ST175, 9.4% in ST233, 6.3% in ST395, 4.9% in ST654, 3.6% in ST298, and 30.0% in others (including 4.0% nontypeable isolates). Thus, more than half of the sequence types of our collection (ST235, ST244, ST175, and ST233; 55.2%) belong to the top 10 P. aeruginosa high-risk clones with worldwide distribution, with ST235 being the most prevalent. All ST235 isolates were positive for the *exoU* gene, while all isolates of ST244, ST175, and ST233 were positive for the *exoS* gene. Of note, no further carbapenemases except VIM and GES were detected (all strains were negative for *bla*_IMP_, *bla*_NDM_, *bla*_GIM_, *bla*_SPM_, and *bla*_KPC_) (see Table S1 in the supplemental material).

### First experiences with cefiderocol in the therapy of infections with MDR P. aeruginosa.

Furthermore, to assess the clinical relevance of the *in vitro* data, we screened our database for cefiderocol treated cases. In the period of 2020 to 2021, eight patients were treated with cefiderocol for targeted antimicrobial therapy. Patients were 58 years old (median, range 18 to 87 years) and had clinical and cultural evidence for infections due to MDR P. aeruginosa with extensive coresistances, except in one neutropenic patient that received cefiderocol as salvage therapy (Table 2). All P. aeruginosa isolates were proven to be cefiderocol susceptible according to EUCAST breakpoints before initiating therapy. Four patients suffered from complicated urinary tract infections (cUTIs), two patients from hospital-acquired pneumonia (HAP), and one patient was treated each for osteomyelitis (petrositis) and bloodstream infection. The bloodstream infection case resulted in sepsis and was caused by complicated intra-abdominal infection (cIAI) during neutropenia. All patients showed microbiological response to cefiderocol therapy, which means that blood cultures, urine cultures, or swabs from the indicated site of infection became P. aeruginosa negative during therapy. Furthermore, all patients showed clinical response to antimicrobial therapy and survived >30 days, except the one patient with septicemia. In 5 cases, infection was caused by *exoU ^+^* P. aeruginosa, while three of them were VIM producing as well (all belonging to the high-risk clone ST235), suggesting an epidemiological dominance of *exoU ^+^*/*bla*_(VIM)_*^+^* above *exoS ^+^*/*bla*_(VIM)_*^+^* isolates at University Hospital Frankfurt (UHF).

**TABLE 2:** Characteristics of patients treated with cefiderocol due to infection with P. aeruginosa^*a*^

Patient	Sex	Age	Infection^*b*^	Resistance type^*c*^	Coresistances^*d*^	Genotype	MLST	Cefiderocol MIC (mg/L)	Microbiological^*e*^ response	30-day survival
1	M	81	Osteomyelitis	MDR	TOB, AM, FOS, CZA, CTB	*exoS* ^−^/*exoU* ^+^, *bla*_VIM_^+^*/bla*_IMP_^−^/*bla*_GES_^−^	ST235	0.5	y	y
2	M	71	cUTI	MDR	ATM, FOS, CZA	*exoS* ^+^/*exoU* ^−^, *bla*_VIM_^−^ *bla*_IMP_^−^/*bla*_GES_^−^	ST179	0.5	y	y
3	M	79	cUTI	MDR	TOB, AM, FOS, CZA, CTB	*exoS* ^−^/*exoU* ^+^, *bla*_VIM_^+^*/bla*_IMP_^−^**/***bla*_GES_^−^	ST235	1.0	y	y
4	M	87	cUTI	MDR	ATM, TOB, AM, FOS, CZA, CTB	*exoS* ^−^/*exoU* ^+^, *bla*_VIM_^+^*/bla*_IMP_^−^/*bla*_GES_^−^	ST235	0.25	y	y
5	M	49	HAP	MDR	ATM, TOB, AM, FOS, CZA, CTB	*exoS* ^−^/*exoU* ^+^, *bla*_VIM_^−^*/bla*_IMP_^−^/*bla*_GES_^−^	n. t.	0.5	y	y
6	M	57	HAP	MDR	ATM, TOB, AM, CZA, CTB	*exoS* ^+^/*exoU*^−^, *bla*_VIM_^−^*/bla*_IMP_^−^/*bla*_GES_^−^	ST166	0.5	y	y
7^*f*^	F	18	cIAI, BSI	CAZ^I^, CIP^I^, AN^I^, TOB^I^	/	*exoS*^+^*exoU*^−,^ *bla*_VIM_^−^*/bla*_IMP_^−^/*bla*_GES_^−^	ST381	0.5	y	n
8	M	36	cUTI	MDR	ATM, TOB, AM, FOS, CZA, CTB	*exoS*^−^/*exoU*^+^, *bla*_VIM_^−^*/bla*_IMP_^−^/*bla*_GES_^−^	ST357	2.0	y	y

a y, yes; n, no; n. t., not typeable (see also Table S3 in the supplemental material).

b Complicated urinary tract infection (cUTI), hospital-acquired pneumonia (HAP), complicated intra-abdominal infection (cIAI), and bloodstream infection (BSI).

c MDR was defined as nonsusceptibility for piperacillin-tazobactam, ceftazidime (CAZ), cefepime, imipenem, meropenem, and ciprofloxacin (CIP). For non-MDR P. aeruginosa of patient 7, substances with (S) susceptibility/(I) susceptibility with increased exposure are indicated (EUCAST-based testing since 2019).

d Coresistances: ceftolozane-tazobactam (CTB), ceftazidime-avibactam (CZA), amikacin (AM), tobramycin (TOB), fosfomycin (FOS), aztreonam (ATM); CTB was recalled from all markets in December 2020 (the unavailability of CTB lasted until February 2022). Note, FOS, AN, and TOB are primarily useful for antipseudomonal combination therapy;

e Defined as a change from P. aeruginosa positive to negative culture from respective infection sites.

*^f^*Cefiderocol treatment in patient 7 was initiated for non-MDR P. aeruginosa due to insufficient clinical response and increasing β-lactam MICs/resistance after 3 weeks of meropenem and 10 days of meropenem/amikacin;

## DISCUSSION

According to WHO, MDR P. aeruginosa, along with A. baumannii and *Enterobacterales*, is one of the top pathogens for which the development of novel antibiotic agents is critical. In the last few years, ceftazidime-avibactam, ceftolozane-tazobactam, and cefiderocol, namely, three new cephalosporins with promising activity against MDR Gram-negative organisms, became available. This study compared the antimicrobial activity of these three drugs against 223 MDR P. aeruginosa, including 71% (158/223) XDR P. aeruginosa. In total, 81 VIM- and 4 GES-5-producing isolates were included resulting in a carbapenemase positivity rate of 38.1% (85/223). Strikingly, most prevalent was the VIM- and ExoU-producing high-risk clone ST235, which is distributed worldwide and associated with epidemics and poor clinical outcome (4).

Activity of ceftazidime-avibactam and ceftolozane-tazobactam against this set of MDR P. aeruginosa was comparable (susceptibility rates were 48.4% and 46.6%, respectively), while cefiderocol was the most active substance with a resistance rate of only 2.7%, making it superior to all other drugs tested (except colistin). In carbapenemase-negative isolates, 75.4%, 76.1%, and 95.7% of isolates were susceptible to ceftolozane-tazobactam, ceftazidime-avibactam, and cefiderocol, respectively. As expected, ceftazidime-avibactam and ceftolozane-tazobactam were inactive against MBL-producing P. aeruginosa, while all were susceptible to cefiderocol. Three of five GES-producing P. aeruginosa isolates were ceftazidime-avibactam susceptible (all with GES-5, known to spare ceftazidime-avibactam) (18).

Of note, this study reports higher resistance rates for ceftazidime-avibactam and ceftolozane-tazobactam than other studies, which often address only carbapenem-resistant P. aeruginosa (CR-PA) with fewer coresistances. However, a precise comparison of resistance rates across studies is generally difficult, as available studies often use different MDR definitions. The Enhancing Rational Antimicrobials against carbapenem-resistant P. aeruginosa (ERACE-PA) study covering a global CR-PA panel reported susceptibility rates of 72% for ceftazidime-avibactam and 63% for ceftolozane-tazobactam (with 33% carbapenemase positivity) (19). The global Study for Monitoring Antimicrobial Resistance Trends (SMART) surveillance program 2018 to 2019 reports ceftolozane-tazobactam susceptibility rates of 87.8% for CR-PA and 76.2% for MDR-PA (carbapenemase prevalence was not reported) (20). Likely, the stringent selection criteria, especially the high XDR and high carbapenemase rate among our P. aeruginosa collection may explain the high ceftazidime-avibactam and ceftolozane-tazobactam resistance rates. In P. aeruginosa, resistances against β-lactams, including carbapenems, often result from overlapping resistance mechanisms, such as OprD loss, *ampC* derepression, and overexpression of efflux pumps, but less frequently from carbapenemases (21). Isolates with decreased *oprD* and increased *mexB* expression exhibit typically lower MICs for ceftolozane-tazobactam than for ceftazidime-avibactam (22, 23). However, P. aeruginosa may develop resistance to ceftolozane-tazobactam and ceftazidime-avibactam, by the expression of AmpC variants (named Pseudomonas-derived cephalosporinases) especially in a derepressed *ampC* background (24–26). Interestingly, in our study, only 39% of MDR P. aeruginosa isolates were susceptible to both ceftolozane-tazobactam and ceftazidime-avibactam, while cross-resistance was found in 44.8% of isolates. As expected, ceftazidime-avibactam and ceftolozane-tazobactam had no activity against VIM-producing phenotypes, while for VIM-negative isolates, susceptibility rates for ceftazidime-avibactam and ceftolozane-tazobactam were significantly higher (76.1% and 75.4%, respectively). Thus, in case of carbapenemase-producing MDR P. aeruginosa, cefiderocol should be the preferred β-lactam choice as the vast majority of isolates are expected to be susceptible, due to its unique mechanism that overcomes decreased membrane permeability, OprD deficiency, and *ampC* derepression. The high *in vitro* activity of cefiderocol and its superiority over ceftazidime-avibactam and ceftolozane-tazobactam demonstrated here are in agreement with results of international surveys. Among the SIDERO-WT-2014 P. aeruginosa study, isolates were 99.9% cefiderocol susceptible. The CANWARD surveillance study reported for MDR/XDR P. aeruginosa a cefiderocol susceptibility rate of 98.3%. Furthermore, 97.3% of XDR P. aeruginosa isolates of the SENTRY Antimicrobial Surveillance Program that covers isolates from United States and Europe were cefiderocol susceptible.

To date, we used cefiderocol for antipseudomonal therapy in 8 patients. Microbiological clearing and clinical treatment outcomes of our patients were most successful in cUTI cases (*n* = 4) compared with those of other infection sites. In agreement, noninferiority of cefiderocol to alternative antibiotic options has been shown in the treatment of cUTI due to MDR organisms (10). Moreover, in the empirical treatment of nosocomial pneumonia, cefiderocol was noninferior to meropenem prolonged infusion (27). The CREDIBLE-CR study that compared cefiderocol versus best available therapy in the treatment of different kind of infections (BSI, sepsis, pneumonia, and cUTI) due to different Gram-negative pathogens (including P. aeruginosa) reports with comparable clinical and microbiological efficacy but higher mortality in the cefiderocol group (11). Thus, administration of cefiderocol requires careful consideration of a patient’s clinical situation and all available alternative antibiotic options. Further outcome studies are required to better assess the clinical effectiveness of cefiderocol. In addition, *in vitro* activity of cefiderocol needs to be continuously monitored to detect shifts in MIC values and resistance rates. The development of resistance may result from diverse mechanisms, including mutations in the iron transport pathway and β-lactamases such as VIM, GIM, GES-6, and PDC-30 (28).

Finally, we have to consider limitations of this study. We only used MTS to test ceftazidime-avibactam, ceftolozane-tazobactam, and cefiderocol that all have been approved for susceptibility testing of P. aeruginosa. For ceftolozane-tazobactam and ceftazidime-avibactam MTS, but not cefiderocol, several studies showed that they correlate well with reference broth microdilution (29–33). For cefiderocol MTS, we found that compared to agar dilution categorical agreement but not essential agreement met the acceptability criterion of ≥90%. Verification of cefiderocol-resistant isolates by BMD confirmed resistance in only two isolates detected by MTS as well (resulting in a very major rate of 0% and a major error rate of 1.8%). However, Albano and colleagues (34) showed that the agar dilution of cefiderocol is error sensitive. As we so far performed multilocus sequence typing analysis, we cannot exclude that this monocentric P. aeruginosa collection may harbor clonal duplicates that derived either from the spread of dominant clones and/or in-hospital nosocomial transmission events. However, no outbreaks with MDR P. aeruginosa were reported during the study period.

In summary, in this study, the overall susceptibility of MDR/XDR P. aeruginosa for ceftolozane-tazobactam, ceftazidime-avibactam, and cefiderocol was 44.6%, 48.4%, and 97.3%, respectively. Thus, in line with recent international studies, cefiderocol was the most active available antipseudomonal β-lactam agent, while further studies are needed to better define its role in clinical practice. Ceftolozane-tazobactam and ceftazidime-avibactam remained fairly active against noncarbapenemase-producing MDR P. aeruginosa (susceptibility around 75%). Therefore, prior empirical ceftazidime-avibactam or ceftolozane-tazobactam therapy verification of the carbapenemase status (e.g., for VIM) may be very valuable at least in a high-prevalent carbapenemase background. Thereby, these data underline the need for continuous surveillance programs in order to monitor (local) resistance and carbapenemase rates of MDR P. aeruginosa as well as the prevalence of P. aeruginosa high-risk clones.

## MATERIALS AND METHODS

### Ethical statement.

A waiver from the local institutional ethical committee approved the microbiological testing of clinical isolates. The evaluation of clinical information within this study is approved by ethical statement number 2021-370.

### Bacterial strains.

A total of 223 multidrug-resistant P. aeruginosa isolates from clinical and screening samples were collected during routine microbiological diagnostics at the University Hospital Frankfurt throughout the years 2013 to 2017 (covering 83% of all MDR P. aeruginosa isolates documented). MDR P. aeruginosa was defined as isolates nonsusceptible for at least piperacillin-tazobactam, ceftazidime, cefepime, imipenem, meropenem, and ciprofloxacin. Species identification was done using Vitek-MS (bioMérieux, Nuertingen, Germany). Antimicrobial susceptibility testing (AST) of P. aeruginosa during routine diagnostics was performed with Vitek 2 using N248 card (bioMérieux) or MIC test strips (MTS) according to recommendations of the Clinical and Laboratory Standards Institute (CLSI) (35). All laboratory procedures were performed under quality-controlled standards (laboratory accreditation according to ISO 15189:2007 standards; certificate number d-ML-13102-01-00). Copy strains from the same patient and year as well as MDR P. aeruginosa isolates from cystic fibrosis patients were excluded.

### Antimicrobial susceptibility testing (AST).

AST of MDR P. aeruginosa for ceftolozane-tazobactam, ceftazidime-avibactam, and cefiderocol was done by MIC gradient strips (Liofilchem, Roseto degli Abruzzi, Italy). MIC strips (MTS) for ceftolozane-tazobactam, ceftazidime-avibactam, and cefiderocol each covered antibiotic concentration ranges of 0.016 mg/L to 256 mg/L. As MTS for testing cefiderocol against P. aeruginosa were approved very recently, their performance was verified for the total strain set against agar dilution. Agar dilution was performed by inoculating 1 μL of a 1:10 dilution of bacterial suspension (0.5 McFarland standard) onto cation-adjusted Mueller-Hinton agar (BD Diagnostics, Heidelberg, Germany) supplemented with 0.03 to 32 mg cefiderocol in 2-fold dilution steps. As conventional laboratory agar media provide an iron-limited environment for bacterial growth, the addition of an iron chelator is not recommended (29, 36). Plates were incubated at 35°C for 16 to 20 h. MIC values of cefiderocol-resistant isolates were verified in triplicate by broth microdilution (BMD) according to the Clinical Laboratory and Standards Institute (CLSI) reference method (37). P. aeruginosa ATCC 27853 was used for quality control (QC), and results were within the QC range throughout this study.

### AST data evaluation.

For all 223 P. aeruginosa isolates, MICs of ceftolozane-tazobactam, ceftazidime-avibactam, and cefiderocol were interpreted according to EUCAST version 12.0 (Table 3). Essential agreement and categorical agreement for cefiderocol MTS were calculated using agar dilution as the reference (acceptability criterion, ≥90%). Essential agreement was defined as the number of isolates with MICs within ± one 2-fold dilution of the comparator method (data were included if in the measurable range). MICs acquired by MTS that fall between the typical log_2_ dilution steps were rounded to the next double dilution step. Very major errors and major errors were evaluated according to CLSI definitions (24). Rates for very major errors (categorization of true-resistant isolates as susceptible by MTS) and major errors (categorization of true-susceptible isolates as resistant by MTS) were calculated according to standard definitions using the number of resistant or susceptible isolates determined by agar dilution (cefiderocol) as the reference.

**TABLE 3:** Breakpoints and QC recommendations for P. aeruginosa (in mg/L)

Agent	MIC^*a*^ of:		Quality control^*b*^ MIC of:	
	≤S	>R		
			Target^*c*^	Range^*d*^
Ceftolozane-tazobactam	4	4	0.5	0.25–1
Ceftazidime-avibactam	8	8	1–2	0.5–4
Cefiderocol	2	2	0.125–0.25	0.06–0.5

a S, susceptible; R, resistant.

b For P. aeruginosa ATCC 27853.

c Calculated by EUCAST.

d From CLSI M100-S30, 2020; all ranges have been validated by EUCAST (version 12.0, 2022, http://www.eucast.org).

### PCR detection of *bla* genes for IMP, VIM, and GES and of genes encoding virulence-associated traits ExoS and ExoU in MDR P. aeruginosa.

All P. aeruginosa isolates were screened for *bla*_VIM_, *bla*_IMP_, and *bla*_GES_ genes by PCR. DNA templates were prepared from bacterial suspension by boiling. PCR amplification of *bla*_VIM_ was performed with primers VIM2004A (5′-GTT TGG TCGCAT ATC GCA AC-3′) and VIM2004B (5′-AAT GCG CAG CAC CAG GATAG-3′) resulting in a 382-bp amplicon (38). IMP-A (5′-GAA GGY GTT TAT GTT CAT AC-3′) and IMP-B (5′-GTA MGT TTCAAG AGT GAT GC-3′) primers, resulting in a 587-bp amplicon, were used for *bla*_IMP_ detection (38). Amplification of *bla*_GES_ was done with primers GES P1 (5′-ATG CGC TTC ATT CAC GCA C-3′) and GES P2 (5′-CTA TTT GTC CGT GCT CAG G-3′), leading to an 846-bp amplicon (39). Identification of carbapenemase type was done by sequencing. The presence of the *exoS* and *exoU* genes was determined by PCR using primers exoS-F (5′-TTG AAG GGA CTC GAC AAG GC-3′) and exoS-R (5′-GCT GTC TGC CCA GGT ACT TT-3′), as well as exoU-F (5′- GCC TTC AGA GCG TCA TAC CT-3′) and exoU-R (5′-GCC AGG GCG ATA CAG AGA G-3′) resulting in a 430-bp and 446-bp amplicon, respectively (this study).

### Sequencing of isolates and processing of sequence data.

DNA of cultured bacteria was extracted using DNeasy UltraClean 96 kit (Qiagen, Venlo, Netherlands). Library preparation and isolates sequencing in this study were either carried out at the Twincore (Center of Clinical and Experimental Infection Research, Hanover, Germany) or were performed by a commercial service provider (Novogene, Cambridge, UK). All isolates were sequenced using Illumina chemistry utilizing a paired-end sequencing strategy of either 2 × 150 bp or 2 × 250 bp.

Postsequencing, a quality assessment of all FastQ files was performed using FastQC version 0.11.8 (40). Remaining partial adapter sequences were removed with Cutadapt (41). *De novo* assembly was carried out using Unicycler version 0.4.8-beta as an optimizer for SPAdes (42). Assembly statistics were assessed using R (43) version 3.4.4 with the package SeqinR (44). As quality criteria, (i) total assembled length of each bacterial isolate had to fall between 6 and 7.5 Mbp, (ii) *N*_90_ values of assemblies had to be above 10 kb, and (iii) the total amount of contigs of an assembly larger than 1 kb had to be below 300.

### Multilocus sequence typing and extended *in silico* search for antimicrobial resistance genes.

The seven-gene multilocus sequence type (MLST) for each P. aeruginosa isolate was determined using MLST (45) version 2.18.0. Typing schemes from the PubMLST database were applied (http://pubmlst.org/paeruginosa/). Identification of additional bacterial antibiotic resistance genes on the nucleotide level was carried out. Assemblies were analyzed against CARD (46) using Abricate (47) version 0.9.8. For these searches, the default 80% similarity and 80% coverage thresholds were applied. In cases where several hits were found for one resistance gene, only the best hit was considered. Similarly, the presence of the genes *exoU* and *exoS* were verified. Reference sequences were obtained from Virulence Factor Database (VFDB) (48).

### Data availability.

Sequence data generated in this study were deposited in the NCBI Sequence Read Archive (SRA) accessible under BioProject PRJNA869680 and the previously uploaded PRJNA526797. SRA accession numbers for all isolates are given in Table S3 in the supplemental material.

## References

[1] Tacconelli E, Carrara E, Savoldi A, Harbarth S, Mendelson M, Monnet DL, Pulcini C, Kahlmeter G, Kluytmans J, Carmeli Y, Ouellette M, Outterson K, Patel J, Cavaleri M, Cox EM, Houchens CR, Grayson ML, Hansen P, Singh N, Theuretzbacher U, Magrini N, Aboderin AO, Al-Abri SS, Awang Jalil N, Benzonana N, Bhattacharya S, Brink AJ, Burkert FR, Cars O, Cornaglia G, Dyar OJ, Friedrich AW, Gales AC, Gandra S, Giske CG, Goff DA, Goossens H, Gottlieb T, Guzman Blanco M, Hryniewicz W, Kattula D, Jinks T, Kanj SS, Kerr L, Kieny M-P, Kim YS, Kozlov RS, Labarca J, Laxminarayan R, Leder K, WHO Pathogens Priority List Working Group. et al. 2018. Discovery, research, and development of new antibiotics: the WHO priority list of antibiotic-resistant bacteria and tuberculosis. Lancet Infect Dis 18:318–327. doi:10.1016/S1473-3099(17)30753-3. 29276051

[2] Botelho J, Grosso F, Peixe L. 2019. Antibiotic resistance in Pseudomonas aeruginosa—mechanisms, epidemiology and evolution. Drug Resist Updat 44:100640. doi:10.1016/j.drup.2019.07.002. 31492517

[3] Reynolds D, Kollef M. 2021. The epidemiology and pathogenesis and treatment of Pseudomonas aeruginosa infections: an update. Drugs 81:2117–2131. doi:10.1007/s40265-021-01635-6. 34743315 PMC8572145

[4] Del Barrio-Tofiño E, López-Causapé C, Oliver A. 2020. Pseudomonas aeruginosa epidemic high-risk clones and their association with horizontally-acquired β-lactamases: 2020 update. Int J Antimicrob Agents 56:106196. doi:10.1016/j.ijantimicag.2020.106196. 33045347

[5] Treepong P, Kos VN, Guyeux C, Blanc DS, Bertrand X, Valot B, Hocquet D. 2018. Global emergence of the widespread Pseudomonas aeruginosa ST235 clone. Clin Microbiol Infect 24:258–266. doi:10.1016/j.cmi.2017.06.018. 28648860

[6] Pelegrin AC, Palmieri M, Mirande C, Oliver A, Moons P, Goossens H, van Belkum A. 2021. Pseudomonas aeruginosa: a clinical and genomics update. FEMS Microbiol Rev 45:fuab026. doi:10.1093/femsre/fuab026. 33970247

[7] Livermore DM, Mushtaq S, Meunier D, Hopkins KL, Hill R, Adkin R, Chaudhry A, Pike R, Staves P, Woodford N, BSAC Resistance Surveillance Standing Committee. 2017. Activity of ceftolozane/tazobactam against surveillance and “problem” Enterobacteriaceae, Pseudomonas aeruginosa and non-fermenters from the British Isles. J Antimicrob Chemother 72:2278–2289. doi:10.1093/jac/dkx136. 28520867 PMC5890766

[8] Yahav D, Giske CG, Grāmatniece A, Abodakpi H, Tam VH, Leibovici L. 2020. New β-lactam-β-lactamase inhibitor combinations. Clin Microbiol Rev 34:e00115-20. doi:10.1128/CMR.00115-20. PMC7667665 33177185

[9] Longshaw C, Manissero D, Tsuji M, Echols R, Yamano Y. 2020. In vitro activity of the siderophore cephalosporin, cefiderocol, against molecularly characterized, carbapenem-non-susceptible Gram-negative bacteria from Europe. JAC Antimicrob Resist 2:dlaa060. doi:10.1093/jacamr/dlaa060. 34223017 PMC8210120

[10] Lee YR, Yeo S. 2020. Cefiderocol, a new siderophore cephalosporin for the treatment of complicated urinary tract infections caused by multidrug-resistant pathogens: preclinical and clinical pharmacokinetics, pharmacodynamics, efficacy and safety. Clin Drug Invest 40:901–913. doi:10.1007/s40261-020-00955-x. PMC7374078 32700154

[11] Bassetti M, Echols R, Matsunaga Y, Ariyasu M, Doi Y, Ferrer R, Lodise TP, Naas T, Niki Y, Paterson DL, Portsmouth S, Torre-Cisneros J, Toyoizumi K, Wunderink RG, Nagata TD. 2021. Efficacy and safety of cefiderocol or best available therapy for the treatment of serious infections caused by carbapenem-resistant Gram-negative bacteria (CREDIBLE-CR): a randomised, open-label, multicentre, pathogen-focused, descriptive, phase 3 trial. Lancet Infect Dis 21:226–240. doi:10.1016/S1473-3099(20)30796-9. 33058795

[12] Yoon E-J, Jeong SH. 2021. Mobile carbapenemase genes in Pseudomonas aeruginosa. Front Microbiol 12:614058. doi:10.3389/fmicb.2021.614058. 33679638 PMC7930500

[13] Horcajada JP, Montero M, Oliver A, Sorlí L, Luque S, Gómez-Zorrilla S, Benito N, Grau S. 2019. Epidemiology and treatment of multidrug-resistant and extensively drug-resistant Pseudomonas aeruginosa infections. Clin Microbiol Rev 32:e00031-19. doi:10.1128/CMR.00031-19. 31462403 PMC6730496

[14] Fischer S, Dethlefsen S, Klockgether J, Tümmler B. 2020. Phenotypic and genomic comparison of the two most common exou-positive Pseudomonas aeruginosa clones, PA14 and ST235. mSystems 5:e01007-20. doi:10.1128/mSystems.01007-20. 33293405 PMC7743143

[15] Ozer EA, Nnah E, Didelot X, Whitaker RJ, Hauser AR. 2019. The population structure of Pseudomonas aeruginosa is characterized by genetic isolation of exoU+ and exoS+ lineages. Genome Biol Evol 11:1780–1796. doi:10.1093/gbe/evz119. 31173069 PMC6690169

[16] Magiorakos A-P, Srinivasan A, Carey RB, Carmeli Y, Falagas ME, Giske CG, Harbarth S, Hindler JF, Kahlmeter G, Olsson-Liljequist B, Paterson DL, Rice LB, Stelling J, Struelens MJ, Vatopoulos A, Weber JT, Monnet DL. 2012. Multidrug-resistant, extensively drug-resistant and pandrug-resistant bacteria: an international expert proposal for interim standard definitions for acquired resistance. Clin Microbiol Infect 18:268–281. doi:10.1111/j.1469-0691.2011.03570.x. 21793988

[17] Sawa T, Shimizu M, Moriyama K, Wiener-Kronish JP. 2014. Association between Pseudomonas aeruginosa type III secretion, antibiotic resistance, and clinical outcome: a review. Crit Care 18:668. doi:10.1186/s13054-014-0668-9. 25672496 PMC4331484

[18] Fraile-Ribot PA, Fernández J, Gomis-Font MA, Forcelledo L, Mulet X, López-Causapé C, Oliver A. 2021. In vivo evolution of GES β-lactamases driven by ceftazidime/avibactam treatment of Pseudomonas aeruginosa infections. Antimicrob Agents Chemother 65:e0098621. doi:10.1128/AAC.00986-21. 34125593 PMC8370190

[19] Gill CM, Aktaþ E, Alfouzan W, Bourassa L, Brink A, Burnham C-AD, Canton R, Carmeli Y, Falcone M, Kiffer C, Marchese A, Martinez O, Pournaras S, Satlin M, Seifert H, Thabit AK, Thomson KS, Villegas MV, Nicolau DP, ERACE-PA Global Study Group. 2021. The ERACE-PA Global Surveillance Program: ceftolozane/tazobactam and ceftazidime/avibactam in vitro activity against a global collection of carbapenem-resistant Pseudomonas aeruginosa. Eur J Clin Microbiol Infect Dis 40:2533–2541. doi:10.1007/s10096-021-04308-0. 34291323 PMC8590662

[20] Karlowsky JA, Lob SH, Young K, Motyl MR, Sahm DF. 2021. Activity of ceftolozane/tazobactam against Gram-negative isolates from patients with lower respiratory tract infections—SMART United States 2018–2019. BMC Microbiol 21:74. doi:10.1186/s12866-021-02135-z. 33676406 PMC7936229

[21] Lister PD, Wolter DJ, Hanson ND. 2009. Antibacterial-resistant Pseudomonas aeruginosa: clinical impact and complex regulation of chromosomally encoded resistance mechanisms. Clin Microbiol Rev 22:582–610. doi:10.1128/CMR.00040-09. 19822890 PMC2772362

[22] Wi YM, Greenwood-Quaintance KE, Schuetz AN, Ko KS, Peck KR, Song J-H, Patel R. 2018. Activity of ceftolozane-tazobactam against carbapenem-resistant, non-carbapenemase-producing Pseudomonas aeruginosa and associated resistance mechanisms. Antimicrob Agents Chemother 62:e01970-17. doi:10.1128/AAC.01970-17. 29133568 PMC5740377

[23] Teo JQ-M, Lim JC, Tang CY, Lee SJ-Y, Tan SH, Sim JH-C, Ong RT-H, Kwa AL-H. 2021. Ceftolozane/tazobactam resistance and mechanisms in carbapenem-nonsusceptible Pseudomonas aeruginosa. mSphere 6:e01026-20. doi:10.1128/mSphere.01026-20. 33504661 PMC7885320

[24] Humphries RM, Ambler J, Mitchell SL, Castanheira M, Dingle T, Hindler JA, Koeth L, Sei K, Hardy D, Zimmer B, Butler-Wu S, Dien Bard J, Brasso B, Shawar R, Dingle T, Humphries R, Sei K, Koeth L. 2018. CLSI Methods Development and Standardization Working Group best practices for evaluation of antimicrobial susceptibility tests. J Clin Microbiol 56:e01934-17. doi:10.1128/JCM.01934-17. 29367292 PMC5869819

[25] Arca-Suárez J, Vázquez-Ucha JC, Fraile-Ribot PA, Lence E, Cabot G, Martínez-Guitián M, Lasarte-Monterrubio C, Rodríguez-Iglesias M, Beceiro A, González-Bello C, Galán-Sánchez F, Oliver A, Bou G. 2020. Molecular and biochemical insights into the in vivo evolution of AmpC-mediated resistance to ceftolozane/tazobactam during treatment of an MDR Pseudomonas aeruginosa infection. J Antimicrob Chemother 75:3209–3217. doi:10.1093/jac/dkaa291. 32728723

[26] Fraile-Ribot PA, Cabot G, Mulet X, Periañez L, Martín-Pena ML, Juan C, Pérez JL, Oliver A. 2018. Mechanisms leading to in vivo ceftolozane/tazobactam resistance development during the treatment of infections caused by MDR Pseudomonas aeruginosa. J Antimicrob Chemother 73:658–663. doi:10.1093/jac/dkx424. 29149337

[27] Wunderink RG, Matsunaga Y, Ariyasu M, Clevenbergh P, Echols R, Kaye KS, Kollef M, Menon A, Pogue JM, Shorr AF, Timsit J-F, Zeitlinger M, Nagata TD. 2021. Cefiderocol versus high-dose, extended-infusion meropenem for the treatment of Gram-negative nosocomial pneumonia (APEKS-NP): a randomised, double-blind, phase 3, non-inferiority trial. Lancet Infect Dis 21:213–225. doi:10.1016/S1473-3099(20)30731-3. 33058798

[28] Karakonstantis S, Rousaki M, Kritsotakis EI. 2022. Cefiderocol: systematic review of mechanisms of resistance, heteroresistance and in vivo emergence of resistance. Antibiotics 11:723. doi:10.3390/antibiotics11060723. 35740130 PMC9220290

[29] Simner PJ, Patel R. 2020. Cefiderocol antimicrobial susceptibility testing considerations: the Achilles’ heel of the Trojan horse? J Clin Microbiol 59:e00951-20. doi:10.1128/JCM.00951-20. 32727829 PMC7771437

[30] Humphries RM, Hindler JA, Magnano P, Wong-Beringer A, Tibbetts R, Miller SA. 2018. Performance of ceftolozane-tazobactam Etest, MIC test strips, and disk diffusion compared to reference broth microdilution for β-lactam-resistant Pseudomonas aeruginosa isolates. J Clin Microbiol 56:e01633-17. doi:10.1128/JCM.01633-17. 29212704 PMC5824066

[31] Daragon B, Fournier D, Plésiat P, Jeannot K. 2021. Performance of disc diffusion, MIC gradient tests and Vitek 2 for ceftolozane/tazobactam and ceftazidime/avibactam susceptibility testing of Pseudomonas aeruginosa. J Antimicrob Chemother 76:2586–2592. doi:10.1093/jac/dkab236. 34245282

[32] Bailey AL, Armstrong T, Dwivedi H-P, Denys GA, Hindler J, Campeau S, Traczewski M, Humphries R, Burnham CA. 2018. Multicenter evaluation of the Etest gradient diffusion method for ceftolozane-tazobactam susceptibility testing of Enterobacteriaceae and Pseudomonas aeruginosa. J Clin Microbiol 56:e00717-18. doi:10.1128/JCM.00717-18. 29976590 PMC6113460

[33] Schaumburg F, Bletz S, Mellmann A, Becker K, Idelevich EA. 2019. Comparison of methods to analyse susceptibility of German MDR/XDR Pseudomonas aeruginosa to ceftazidime/avibactam. Int J Antimicrob Agents 54:255–260. doi:10.1016/j.ijantimicag.2019.05.001. 31071465

[34] Albano M, Karau MJ, Schuetz AN, Patel R. 2020. Comparison of agar dilution to broth microdilution for testing in vitro activity of cefiderocol against Gram-negative bacilli. J Clin Microbiol 59:e00966-20. doi:10.1128/JCM.00966-20. 32967901 PMC7771473

[35] CLSI. Performance standards for antimicrobial susceptibility testing—information supplement. CLSI document M100-S13 to M100-S17. CLSI, Wayne, PA.

[36] Critchley IA, Basker MJ. 1988. Conventional laboratory agar media provide an iron-limited environment for bacterial growth. FEMS Microbiology Lett 50:35–39. doi:10.1111/j.1574-6968.1988.tb02907.x.

[37] CLSI. 2022. Performance standards for antimicrobial susceptibility testing, 32nd ed, M100. CLSI, Wayne, PA.

[38] Pitout JDD, Gregson DB, Poirel L, McClure J-A, Le P, Church DL. 2005. Detection of Pseudomonas aeruginosa producing metallo-beta-lactamases in a large centralized laboratory. J Clin Microbiol 43:3129–3135. doi:10.1128/JCM.43.7.3129-3135.2005. 16000424 PMC1169086

[39] Wang C, Cai P, Chang D, Mi Z. 2006. A Pseudomonas aeruginosa isolate producing the GES-5 extended-spectrum beta-lactamase. J Antimicrob Chemother 57:1261–1262. doi:10.1093/jac/dkl116. 16617065

[40] Andrews S, Lindenbaum P, Howard B, Ewels P. 2010. FastQC: a quality control tool for high throughput sequence data. https://www.bioinformatics.babraham.ac.uk/projects/fastqc/. Accessed 09 July 2019.

[41] Martin M. 2011. Cutadapt removes adapter sequences from high-throughput sequencing reads. EMBnet j 17:10. doi:10.14806/ej.17.1.200.

[42] Prjibelski A, Antipov D, Meleshko D, Lapidus A, Korobeynikov A. 2020. Using SPAdes de novo assembler. Curr Protoc Bioinformatics 70:e102. doi:10.1002/cpbi.102. 32559359

[43] R Core Team. 2018. R: a language and environment for statistical computing. R Foundation for Statistical Computing, Vienna, Austria. https://www.r-project.org/.

[44] Charif D, Lobry JR. 2007. SeqinR 1.0–2: a contributed package to the R project for statistical computing devoted to biological sequences retrieval and analysis, 207–232. *In* Structural approaches to sequence evolution. Springer, Berlin, Heidelberg. doi:10.1007/978-3-540-35306-5_10.

[45] Seemann T. Abricate. https://github.com/tseemann/snp-dist. Accessed 24 June 2020.

[46] Jia B, Raphenya AR, Alcock B, Waglechner N, Guo P, Tsang KK, Lago BA, Dave BM, Pereira S, Sharma AN, Doshi S, Courtot M, Lo R, Williams LE, Frye JG, Elsayegh T, Sardar D, Westman EL, Pawlowski AC, Johnson TA, Brinkman FSL, Wright GD, McArthur AG. 2017. CARD 2017: expansion and model-centric curation of the comprehensive antibiotic resistance database. Nucleic Acids Res 45:D566–D573. doi:10.1093/nar/gkw1004. 27789705 PMC5210516

[47] Seemann T. Mlst. https://github.com/tseemann/mlst. Accessed 28 January 2020.

[48] Chen L, Zheng D, Liu B, Yang J, Jin Q. 2016. VFDB 2016: hierarchical and refined dataset for big data analysis–10 years on. Nucleic Acids Res 44:D694–D697. doi:10.1093/nar/gkv1239. 26578559 PMC4702877

